# Waddington’s Landscapes in the Bacterial World

**DOI:** 10.3389/fmicb.2021.685080

**Published:** 2021-06-04

**Authors:** María A. Sánchez-Romero, Josep Casadesús

**Affiliations:** Departamento de Genética, Facultad de Biología, Universidad de Sevilla, Sevilla, Spain

**Keywords:** phenotypic heterogeneity, noise, bistability, lineage formation, DNA methylation

## Abstract

Conrad Waddington’s epigenetic landscape, a visual metaphor for the development of multicellular organisms, is appropriate to depict the formation of phenotypic variants of bacterial cells. Examples of bacterial differentiation that result in morphological change have been known for decades. In addition, bacterial populations contain phenotypic cell variants that lack morphological change, and the advent of fluorescent protein technology and single-cell analysis has unveiled scores of examples. Cell-specific gene expression patterns can have a random origin or arise as a programmed event. When phenotypic cell-to-cell differences are heritable, bacterial lineages are formed. The mechanisms that transmit epigenetic states to daughter cells can have strikingly different levels of complexity, from the propagation of simple feedback loops to the formation of complex DNA methylation patterns. Game theory predicts that phenotypic heterogeneity can facilitate bacterial adaptation to hostile or unpredictable environments, serving either as a division of labor or as a bet hedging that anticipates future challenges. Experimental observation confirms the existence of both types of strategies in the bacterial world.

## Introduction

During differentiation of tissues in multicellular eukaryotes, genetically identical cells diversify into cell types that differ in both their morphology and their physiology. In the mid-twentieth century, C. H. Waddington envisioned eukaryotic developmental pathways as a series of ridges and valleys traversed by cells on their way to differentiation (Waddington, 1957). Cell differentiation involving change of form is also found in certain prokaryotic species. Well-known examples include the formation of heterocysts in filamentous cyanobacteria (Muro-Pastor and Hess, 2012), sporulation in *Bacillus subtilis* (Khanna et al., 2020), differentiation of nitrogen-fixing bacteroids in *Rhizobium* spp. (Kondorosi et al., 2013), asymmetric cell division in *Caulobacter* (Collier, 2019), and formation of fruiting bodies by myxobacteria (Munoz-Dorado et al., 2016). In other cases, however, cell differentiation occurs without visible morphological change. In the last few decades, the study of bacterial cell variants has been facilitated by growing interest in bacterial multicellularity (Shapiro, 1998) and by technical upturn in single-cell analysis technologies (Bernander et al., 1998; Meyer and Dworkin, 2007; Kreibich and Hardt, 2015; Scheler et al., 2019).

Phenotypic heterogeneity in a bacterial population can be the consequence of chemical communication, leading to a heterogeneous response at the single-cell level. For instance, differentiation of cyanobacterial heterocysts seems to respond to gradients of activator and inhibitor molecules along the cyanobacterial filament (Muro-Pastor and Hess, 2012). In other cases, phenotypic heterogeneity arises without the involvement of environmental cues, and the underlying mechanisms are diverse. Genetic mechanisms include site-specific recombination (Scott and Simon, 1982; Reyes Ruiz et al., 2020), slipped-strand mispairing at tracts of repetitive DNA sequences (Moxon et al., 2006), and amplification of specific genome regions (Belikova et al., 2020; Tomanek et al., 2020). As described below, cell diversification into two or more phenotypic states can also be driven by nongenetic mechanisms, such as propagation of feedback loops (Ferrell, 2002) and formation of DNA methylation patterns (Sanchez-Romero and Casadesus, 2020).

Differentiation of bacterial subpopulations can be interpreted as the manifestation of two different strategies: division of labor and bet hedging (Veening et al., 2008; Lambert and Kussell, 2014). Division of labor is a cooperative activity that increases the fitness of the subpopulations if they coexist (Zhang et al., 2016). Illustrative examples of division of labor have been described in biofilms (van Gestel et al., 2015; Dragos et al., 2018). In bet hedging, a population with more than one phenotype performs better in a changing environment than a population with a homogeneous phenotype, and the variance in offspring numbers across generations is minimized (Gillespie, 1974; de Jong et al., 2011; Schreiber et al., 2016). Bet hedging has been shown to produce subpopulations tolerant to antibacterial agents (Adam et al., 2008; Hernandez et al., 2012; Dewachter et al., 2019) or resistant to bacteriophages (Cota et al., 2015; Turkington et al., 2019).

Natural selection of phenotypic heterogeneity, especially if it involves a bet-hedging strategy, is a controversial notion in classical Darwinism because it involves group selection, which has been traditionally considered a weak evolutionary force (Leigh, 2010). This view is however countered by game theory (Kussell and Leibler, 2005; Wolf et al., 2005; Kussell, 2013).

## Sources of Phenotypic Differences in Isogenic Bacterial Cells

Events in cellular physiology involve random encounters between molecules, some of which are present in small numbers. As a consequence, a certain degree of stochasticity exists in many biochemical transactions (Kaern et al., 2005; Sanchez et al., 2013). A physiological event where stochasticity is well known is transcription initiation, which can show differences from one cell to another. As a consequence, cells with distinct transcriptional profiles can be produced in isogenic subpopulations of bacteria (Silva-Rocha and de Lorenzo, 2010). Changes in gene copy number during the bacterial cell cycle and cell-to-cell differences in translation efficiency are additional sources of stochasticity in gene expression (Kaern et al., 2005).

Noise can be sufficient to produce phenotypic heterogeneity in a bacterial population. Because of the finite number effect, a small difference in the number of molecules can produce or not a signal with physiological significance (Kaern et al., 2005). Thresholds are therefore crucial in noisy systems to produce a “meaningful” signal (Anderson, 1972). When distinct gene expression patterns generated by noise are propagated by feedback loops, the bacterial population splits into subpopulations, a phenomenon known as multistability (Thomas and Kaufman, 2001).

Most examples of multistability validated by experimental analysis involve two phenotypic states only (bistability), producing cells with high and low expression of specific genes or gene networks (ON and OFF cells) (Laurent et al., 2005; Dubnau and Losick, 2006). In some cases, formation of cell variants is not stochastic but deterministic, and ON and OFF cells show nonlinear gene expression patterns that do not arise from noise (Casadesus and Low, 2013). Whatever their origin, bistable states can be transmitted to the progeny either by a positive feedback loop or by a double-negative feedback loop (Casadesus and D’Ari, 2002; Ferrell, 2002). When reversion of a bistable state occurs in a programmed manner, bistability is called phase variation (van der Woude, 2011).

## Bistable States Propagated by Feedback Loops

Examples of bacterial bistable systems sustained by feedback loops are reviewed below. Some have been chosen because of their historic relevance; other choices may be arbitrary. A rich literature on the subject exists, including comprehensive reviews (Laurent et al., 2005; Dubnau and Losick, 2006; Veening et al., 2008; Casadesus and Low, 2013; Ackermann, 2015; Weigel and Dersch, 2018; Schroter and Dersch, 2019).

### Bistability in the *lac* Operon

**Figure 1:**
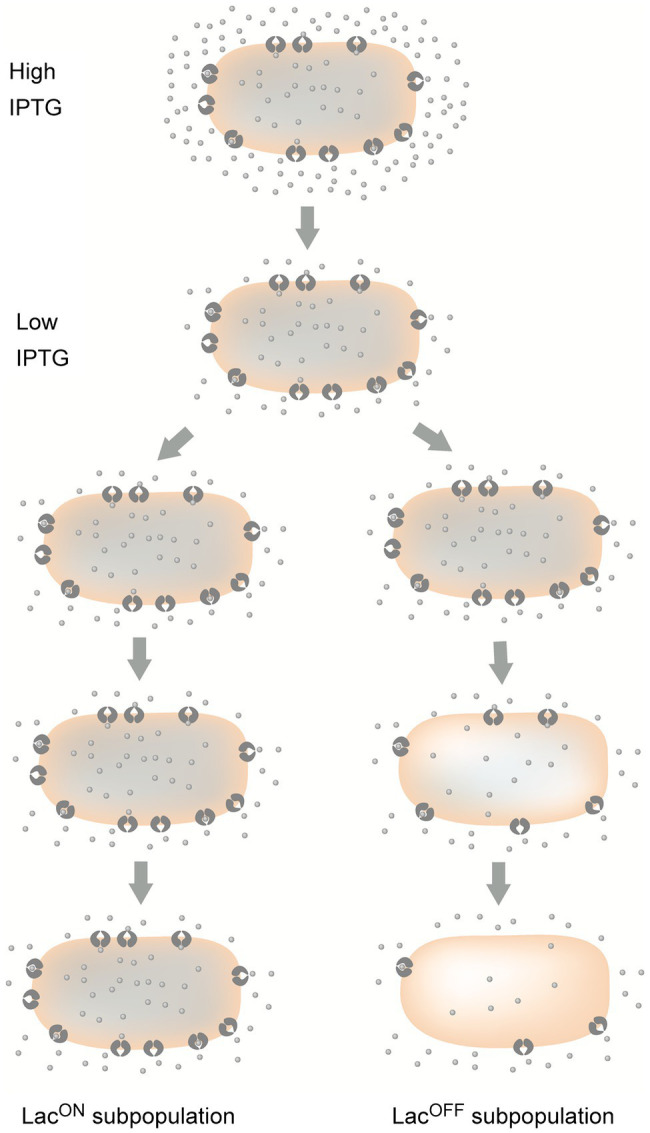
Bifurcation of *lac* operon expression into ON and OFF states after transfer of an induced culture to lower and intermediate levels of the inducer, IPTG. LacI permease molecules are shown inserted in the cytoplasmic membrane. For simplification, the cell wall and the outer membrane are not shown. Dots represent IPTG molecules.

An example of bistability propagated by a positive feedback loop was described in the *lac* operon of *Escherichia coli* more than six decades ago (Novick and Weiner, 1957). Isopropyl-β-D-1-thio-galactoside (IPTG) is a gratuitous (noncatabolizable) inducer that derepresses the *lac* operon if added to the bacterial culture at high concentrations. At low concentrations, IPTG is unable to induce a naïve culture. However, if an induced culture is transferred to a culture medium containing a low concentration of IPTG, a subpopulation of cells remains in the induced state (Novick and Weiner, 1957). Maintenance of Lac^ON^ cells occurs because they have a high level of β-galactoside permease in their membrane. A high level of permease concentrates IPTG inside the cell, and a high concentration of IPTG induces a high level of permease synthesis (Novick and Weiner, 1957; Laurent et al., 2005). In certain cells, however, a decrease in the internal concentration of inducer (which may occur, for instance, during cell elongation or after cell division) reduces permease synthesis, which in turn causes a further reduction in the internal concentration of IPTG, driving the cell toward the Lac^OFF^ state (Figure 1). The overall consequence is that a fully induced population bifurcates into two bistable states: Lac^ON^ and Lac^OFF^ (Novick and Weiner, 1957; Casadesus and D’Ari, 2002; Laurent et al., 2005). The positive feedback loop in this system is that a high permease level concentrates inducer inside the cell, and a high internal level of inducer produces a high level of permease. A potential benefit of this loop may be to drain pools of metabolizable β-galactosides by maintaining high levels of permease when the inducer concentration decreases. Otherwise, a certain amount of β-galactoside might be left unused.

An increased error rate during transcription, caused by mutations that reduce transcriptional fidelity, can trigger switching of the *lac* operon from OFF to ON in the presence of suboptimal concentrations of inducer (Gordon et al., 2009). A decrease in the level of functional LacI repressor below a critical threshold permits transcriptional activation in certain cells (Gordon et al., 2009; Satory et al., 2011). Synthesis of permease then creates a positive feedback loop that maintains the ON state. As in other bistable systems, a small number of LacI repressor molecules per cell (~10) is crucial to make the system noisy (Gordon et al., 2009).

### Competence Development in *Bacillus subtilis*

**Figure 2:**
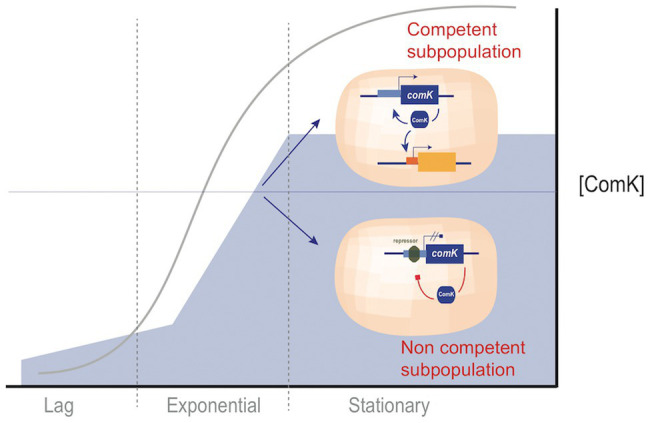
Acquisition of competence by a *Bacillus subtilis* subpopulation. The fate of individual cells is decided at a critical moment in which ComK levels are intermediate and noisy. Above a threshold level, ComK drives the bacterial cell toward competence. Below the threshold level, repressors prevent ComK synthesis, and the cell does not become competent. Under laboratory conditions, the decision is taken when the culture enters stationary phase.

When a *B. subtilis* culture approaches stationary phase, a fraction of cells acquire competence, a physiological state that enables DNA uptake (Dubnau and Losick, 2006). A crucial factor for the development of competence is accumulation of ComK, a transcription factor that activates genes required for DNA uptake as well as the *comK* gene itself (van Sinderen et al., 1995). During exponential growth, ComK is synthesized but degraded. When the culture approaches stationary phase, a quorum-sensing-related factor stabilizes ComK (Magnuson et al., 1994; Turgay et al., 1998). At that moment, a competition starts between several repressors and ComK for binding regulatory regions at the *comK* promoter (Hoa et al., 2002; Hamoen et al., 2003). Binding of ComK initiates a positive feedback loop, leading to increased synthesis of ComK and subsequent transcription of competence genes. Binding of the repressors inhibits *comK* expression and prevents competence. A crucial property for bifurcation of the population into two subpopulations is that the level of ComK in individual cells fluctuates, generating stochastic noise. When the ComK level reaches a threshold in a *B. subtilis* cell, a quantitative difference becomes qualitative: the ComK positive feedback loop is activated, and competence is acquired (Smits et al., 2005, 2006; Dandach and Khammash, 2010). Development of competence thus occurs in cells that undergo a small but critical increase in ComK concentration. In turn, c*omK* is repressed in cells in which the ComK level remains below the threshold, and they do not acquire competence (Smits et al., 2006) (Figure 2).

### Lysis and Lysogeny in Bacteriophages

**Figure 3:**
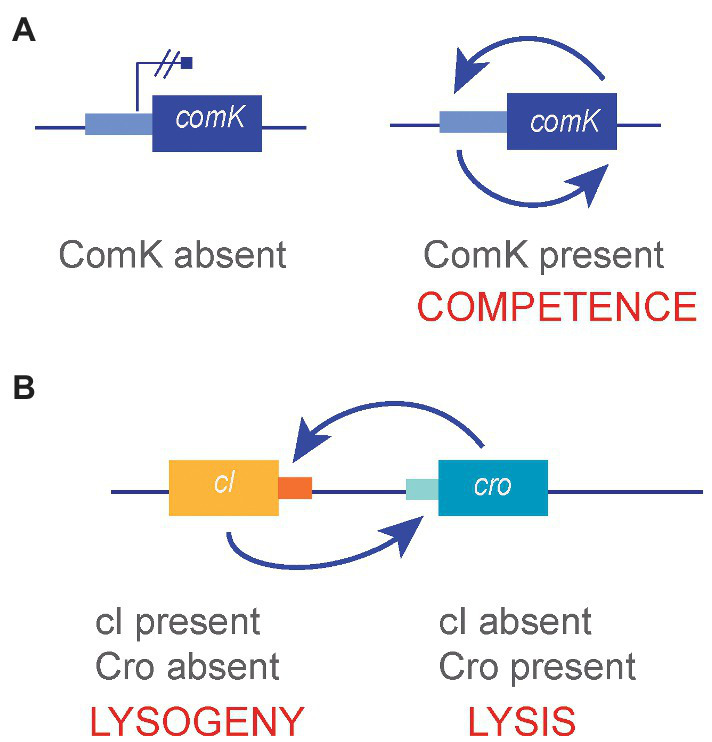
**(A)** Competence development in *B. subtilis*, an example of bistability produced by a positive feedback loop. **(B)** The lysis/lysogeny decision in bacteriophage lambda, an example of bistability produced by a double-negative feedback loop.

Infection of *E. coli* by the temperate bacteriophage lambda can follow two developmental programs: lysis of the bacterial cell and lysogeny. Although the decision is influenced by the physiological state of the cell and by environmental cues, the fate of individual infections is unpredictable and may be considered stochastic (Johnson et al., 1981; Casadesus and D’Ari, 2002; Munsky and Khammash, 2010). Phage lambda has two repressors, CI and Cro, each of which represses the expression of the other. At the onset of infection, both repressors are produced, and the lysis-lysogeny decision may be viewed as a race: The repressor that first occupies specific regulatory DNA sites in the lambda genome will repress the synthesis of its antagonist (Johnson et al., 1981). If the winner is Cro, synthesis of CI will be repressed, and lambda will lyse the host cell (Figure 2). If the winner is CI, synthesis of Cro will be repressed, and lambda will lysogenize the cell (Johnson et al., 1981). Note that the outcomes of a positive feedback loop and a double-negative feedback loop are analogous (Casadesus and D’Ari, 2002; Ferrell, 2002). In the case of lambda, preventing the synthesis of Cro by CI is equivalent to positive autoregulation of *cI* gene expression, and vice versa (Figure 3).

In *E. coli* lysogens for Shiga toxin phages, only a fraction of cells enter the lytic cycle upon prophage induction. This dual strategy may prevent extinction of the bacterial population, at the same time permitting that the phage population generated by induction can introduce the capacity to produce Shiga toxin into new hosts (Imamovic et al., 2016).

### Contribution of Phenotypic Heterogeneity to Antibiotic Tolerance

Stochastic fluctuations in the expression of critical genes can produce bacterial cells that are able to survive in the presence of an antibiotic (Adam et al., 2008). For instance, stochastic activation of the multiple antibiotic resistance activator MarA confers multidrug tolerance in *E. coli* (El Meouche et al., 2016). In *Salmonella enterica*, plating of a batch culture on a lethal (but not extremely high) concentration of kanamycin provides two types of kanamycin-resistant isolates. Some are stable, produced by mutation. Other isolates, however, revert to kanamycin sensitivity upon nonselective growth, indicating a nongenetic origin for the kanamycin-tolerant phenotype. A factor that contributes to tolerance is the formation of a subpopulation of cells that contain reduced levels of the OmpC porin in the outer membrane (Sanchez-Romero and Casadesus, 2014). Expression of *ompC* is noisy, and cells with low OmpC protein in the outer membrane can withstand kanamycin. In the presence of kanamycin, activation of the RpoE-dependent stress response downregulates *ompC* expression (Woods and McBride, 2017). The resulting feedback loop sustains and/or amplifies the cellular state that initially permitted survival, and a kanamycin-tolerant subpopulation is produced (Sanchez-Romero and Casadesus, 2014).

Resistance to fluoroquinolones also has nongenetic components. For instance, the activity of the AcrAB-TolC efflux pump increases the minimal inhibitory concentration of nalidixic acid in *S. enterica* isolates that carry gyrase mutations. Because individual *Salmonella* cells display different levels of *acrAB* expression, the bacterial population is heterogeneous and includes cells with high AcrAB-mediated efflux (Sanchez-Romero and Casadesus, 2014). These cells have reduced growth rate, which can be regarded as a toll for the acquisition of nongenetic resistance (Motta et al., 2015). An inverse correlation between growth and antibiotic tolerance may be common (Claudi et al., 2014).

Nonmutational tolerance to antibiotics is also found in persisters, subpopulations of cells that adopt a dormant state upon decrease or arrest of growth and metabolism (Balaban, 2011; Fisher et al., 2017). Persistence is a reversible epigenetic state (Balaban et al., 2004). Persisters were first described in *Staphylococcus aureus* and more recently in other bacterial pathogens. Especially relevant for human health is the role of persisters in asymptomatic carriage of *Mycobacterium tuberculosis* and other pathogens that cause latent infection (Rhen et al., 2003). Various mechanisms have been proposed to produce persisters (e.g., toxin-antitoxin control, metabolic regulation, and ppGpp-dependent stringent response). The lack of an accepted model may merely reflect the involvement of multiple mechanisms. In fact, persisters of a given species often belong to several phenotypic classes (Dhar and McKinney, 2007; Hofsteenge et al., 2013; Putrins et al., 2015).

### DNA Repair Heterogeneity

The SOS regulon, a bacterial gene network responsive to DNA damage, is under the control of the LexA transcriptional repressor (Baharoglu and Mazel, 2014). In the presence of a DNA damaging agent, LexA is degraded and SOS genes are turned on. However, SOS activation is also observed in a subpopulation of *E. coli* cells during normal growth (McCool et al., 2004; Pennington and Rosenberg, 2007; Kamensek et al., 2010). SOS activation under such conditions is triggered by endogenous DNA-damaging compounds produced by normal metabolism (Xia et al., 2019; Yang et al., 2019). In fact, spontaneous DNA strand breakage is detected in a subset of cells during normal growth (Pennington and Rosenberg, 2007). Heterogeneous activation of the SOS system under apparently optimal growth conditions has been also described in *M. tuberculosis* (Manina et al., 2019).

Heterogeneous expression may be a common feature of DNA repair systems (Vincent and Uphoff, 2020). As a consequence, repair of DNA lesions by the adaptive response to DNA alkylation damage may be accompanied by an increase in the mutation rate in individual cells (Uphoff et al., 2016). Antibiotic-induced activation of the RpoS-dependent general stress response can likewise increase the mutation rate in an *E. coli* subpopulation (Pribis et al., 2019). Variation of mutation rates in response to environmental factors is an old prediction of population genetics (Fitch, 1982) and has been validated by experimental studies (Oliver et al., 2000; Saint-Ruf and Matic, 2006; Pribis et al., 2019). Formation of small colony variants of pathogenic bacteria in animal cells and tissues may be also a manifestation of increased mutation rates (Proctor et al., 1994; Cano et al., 2003; Nelson et al., 2010). The notion of stress-induced mutation has raised concerns about mutational burden (Roth et al., 2006). However, such arguments can be lessened if the mutation rate increases only in a subpopulation of cells.

### Bistable States in Host-Pathogen Interactions

Formation of cell variants in bacterial pathogens has long been recognized as a strategy for evasion of the immune system (Finlay and McFadden, 2006). In addition, cases of phenotypic heterogeneity whose adaptive value does not seem related to immune evasion have been described.

In the opportunistic pathogen *Pseudomonas aeruginosa*, a positive feedback loop involving the transcriptional regulator BexR activates the expression of the so-called BexR regulon, which includes the virulence-related *aprA* gene and other loci of unknown function (Turner et al., 2009). In addition, BexR shows positive autoregulation (Turner et al., 2009). Similar to the *B. subtilis* ComK system, bistable BexR expression is the consequence of noisy, low-level BexR synthesis, followed by autogenous amplification of the BexR level in cells that produce BexR above a critical threshold (Turner et al., 2009). A difference, however, is that competence is acquired by 10% of *B. subtilis* cells (Dubnau and Losick, 2006) while the BexR feedback loop is activated in 0.004% cells only (Turner et al., 2009).

In *Yersinia pseudotuberculosis*, bistable synthesis of the virulence regulator RovA can be viewed as a bet-hedging strategy that preadapts the bacterial population to the changing conditions encountered during early and late stages of infection (Nuss et al., 2016; Weigel and Dersch, 2018). RovA bistability has at least two sources. Activation of *rovA* transcription by RovA is noisy, and a feedback loop of autogenous activation is triggered in cells where RovA reaches a critical threshold. In addition, posttranscriptional control contributes to bistability: a conformational change in a dimerization domain reduces the RovA DNA-binding capacity and increases proteolytic degradation, thus driving the system toward the OFF state. Control of the ratio of ON/OFF cells can be further modulated by two-component systems and global regulators, adjusting the expression of virulence determinants during different stages of infection and in different tissues (Weigel and Dersch, 2018).

Temporal bistability also modulates virulence in *Vibrio cholerae*. At a late stage of animal infection, *V. cholerae* populations bifurcate into two subpopulations, one of which turns off virulence genes while the other remains virulent (Nielsen et al., 2010). The existence of a highly infectious subpopulation in the stools of cholera patients may contribute to *V. cholerae* dissemination. Bifurcation is reversible, and a bistable switch enables or disables the formation of a feedback loop that controls the expression of ToxT, the master regulator of virulence gene expression (Nielsen et al., 2010).

The Gram positive pathogen *S. aureus* causes acute and chronic infections, and the infection outcome is controlled by a quorum-sensing system called Agr (Recsei et al., 1986; Benson et al., 2011). This system shows bistability, with concomitant formation of Agr^OFF^ and Agr^ON^ subpopulations specialized in planktonic and biofilm-associated lifestyles, respectively (García-Betancur et al., 2017). The Agr^ON^ cell lineage, specialized in chronic infection, is produced by a positive feedback loop that activates the expression of biofilm genes in cells where the phosphorylated form of the transcription factor AgrA is present above a critical concentration. Below this threshold, the cells remain Agr^OFF^ and form a subpopulation with acute infection capacity including toxin secretion (García-Betancur et al., 2017).

Another threshold-based decision controls phenotypic heterogeneity in *Xenorhabdus nematophila*, a Gram-negative bacterium used in biological pest control. *X. nematophila* lives a double life, as a pathogen of insects and a mutualist of nematodes that transmit the pathogen to insects. Formation of mutualistic and virulent cell variants is under the control of the transcriptional regulator Lrp, which controls transcription of hundreds of genes (Hussa et al., 2015). The level of Lrp shows cell-to-cell variation, and high Lrp levels promote mutualism, while low Lrp levels promote virulence. As infected nematodes age, a decrease in the Lrp level enhances virulence, anticipating exposure to the insect host (Cao and Goodrich-Blair, 2020). Interestingly, *lrp* mutants, which show a growth advantage at late stages of infection, have reduced virulence and impaired transmission to insects (Cambon et al., 2019), a feature that illustrates how nonmutational variation can be advantageous over mutation.

In *S. enterica*, expression of pathogenicity island 1 (SPI-1) is bimodal, and virulence determinants are secreted by SPI-1^ON^ cells only (Sturm et al., 2011; Arnoldini et al., 2014). Synthesis and/or activity of the type III secretion system (T3SS) slows down the growth of SPI-1^ON^ cells. However, inflammation triggered by the T3SS generates electron acceptors that provide a growth advantage to *Salmonella* over the intestinal microbiota (Diard et al., 2013), a benefit for both SPI-1^ON^ and SPI-1^OFF^ cells. Furthermore, fast growth makes SPI-1^OFF^ cells able to outcompete *Salmonella* avirulent variants (Diard et al., 2013). Epithelial cell invasion by SPI-1^OFF^ cells may extend outcompetition to the intracellular environment, contributing to prevent takeover of the population by avirulent mutants (Sanchez-Romero and Casadesus, 2018).

Additional bifurcations occur during *Salmonella* infection. Bistable expression of *myo*-inositol utilization genes may help to overcome nutrient limitation in the intestine and can be viewed as a bet-hedging strategy (Muller et al., 2019). Epithelial cells infected by *Salmonella* contain either large or small numbers of bacteria, a distribution that suggests bimodality (Garcia-Del Portillo, 2008). During systemic infection, the *Salmonella* population splits into two subpopulations inside macrophages, and one subpopulation multiplies while the other enters a dormant-like state (Helaine et al., 2010).

Colonization of the gall bladder by *Salmonella* provides another example of lineage formation. The bile-laden gall bladder is a harsh environment for bacteria because bile salts are bactericidal (Urdaneta and Casadesus, 2017). *Salmonella* survival in the gall bladder may be facilitated by bifurcation of the population into one lineage that invades the gall bladder epithelium (Menendez et al., 2009), while another lineage remains in the gall bladder lumen. Further diversification occurs if gallstones are present, because *Salmonella* is able to form biofilms on gallstones (Prouty et al., 2002; Crawford et al., 2010). Survival of planktonic cells in the gall bladder lumen may additionally involve phenotypic heterogeneity associated with noisy activation of the RpoS-dependent general stress response in certain cells (Urdaneta et al., 2019).

## Bistable Switches Under DNA Methylation Control

DNA methylation has multiple roles in bacterial physiology, including the control of lineage formation (Casadesus and Torreblanca, 1996; Marinus, 1996; Lobner-Olesen et al., 2005; Wion and Casadesus, 2006; Low and Casadesus, 2008; Vasu and Nagaraja, 2013; Adhikari and Curtis, 2016; Mouammine and Collier, 2018). Formation of cell variants under DNA methylation control may be an especially robust mechanism for subpopulation formation because inheritance of DNA methylation patterns permits faithful transmission of transcriptional states across generations. Furthermore, unlike noise-based switches, the architecture and the DNA methylation state of the regulatory region determine the switching frequencies, thereby producing subpopulations of constant sizes (Casadesus and Low, 2006). In certain cases, the subpopulation sizes can be additionally modulated by cellular regulators responsive to environmental cues (Casadesus and Low, 2006; Sanchez-Romero and Casadesus, 2020).

The widespread involvement of DNA methylation in bacterial pathogenesis (Marinus and Casadesus, 2009; Kumar and Rao, 2013) and the recent development of a DNA sequencing procedure that permits genome-wide detection of N^6^-methyl-adenine (SMRT sequencing) (Flusberg et al., 2010) has given a fresh impulse to the study of DNA methylation in bacterial genomes (Davis et al., 2013). Among other interesting outcomes, SMRT sequencing has broadened our knowledge of the distribution of DNA methylation in bacterial genomes (Blow et al., 2016) and has provided novel examples of bistable loci under DNA methylation control (Sanchez-Romero et al., 2020).

### Control of Lineage Formation by DNA Adenine Methylation

In gammaproteobacteria, formation of DNA adenine methylation patterns (combinations of methylated and nonmethylated GATC sites) provides a mechanism for transmission of epigenetic states to the offspring (Wion and Casadesus, 2006). Nonmethylated sites are often part of clusters of GATCs located within binding sites for transcriptional regulators and are flanked by DNA sequences that reduce the processivity of the Dam methylase (Wion and Casadesus, 2006; Sanchez-Romero and Casadesus, 2020). Binding of the cognate protein hinders Dam methylase activity, generating nonmethylated GATCs after two rounds of DNA replication. Nonmethylation persists as long as the transcription factor remains bound to its cognate sequence, and the methylation pattern can be inherited by daughter cells. However, every DNA replication round provides a window of opportunity to change the DNA methylation pattern of the regulatory region, switching transcription from OFF to ON and vice versa. A paradigm of Dam-dependent epigenetic control is the *pap* operon of uropathogenic *E. coli*, whose workings were brilliantly deciphered by David Low and co-workers in the 1990s and early 2000s (van der Woude et al., 1996; Hernday et al., 2004). The *pap* operon encodes fimbriae that permit adherence to the epithelium of the urinary tract (van der Woude et al., 1996), and formation of Pap^OFF^ and Pap^ON^ lineages may be interpreted as a division of labor: Only the fimbriated subpopulation can colonize the urinary tract, but the nonfimbriated subpopulation makes it possible by avoiding immune system alert. An example of Dam-dependent locus involved in bet hedging is provided by the *Salmonella opvAB* operon, which produces a lineage of cells resistant to bacteriophages at the expense of reducing virulence (Cota et al., 2015).

**Figure 4:**
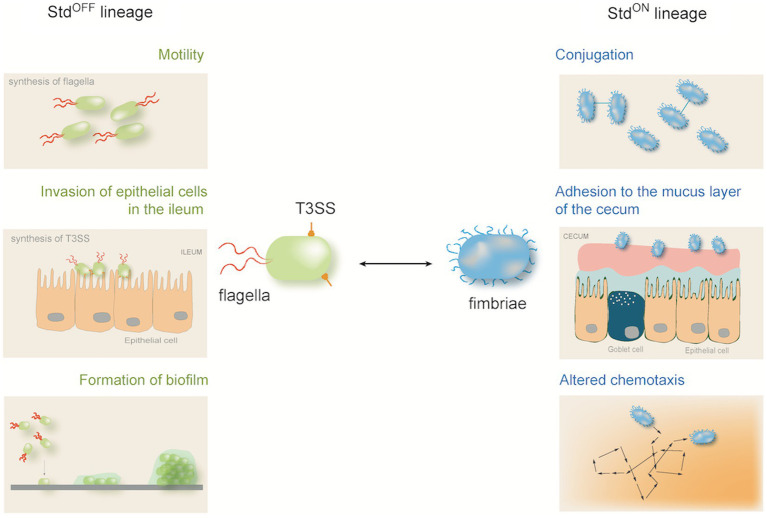
Subpopulation formation under the control of the Std pleiotropic switch in *Salmonella enterica* serovar Typhimurium. In addition to fimbriae, the *std* operon encodes transcriptional regulators that activate or repress hundreds of genes. As a consequence, the Std^OFF^ and Std^ON^ lineages differ in multiple phenotypic traits.

Additional genes and operons under Dam methylation control have been described in *E. coli* and *Salmonella*, each with particular traits and switching frequencies (Sanchez-Romero and Casadesus, 2020). An example that stands out because of its unusual pleiotropy is the *Salmonella std* operon, which encodes fimbriae for adhesion to the mucus layer of the cecum in the large intestine (Suwandi et al., 2019). In addition to fimbrial components, the *std* operon encodes transcriptional regulators that control the expression of hundreds of genes (Garcia-Pastor et al., 2018). Std^OFF^ and Std^ON^ cells thus differ in multiple phenotypic traits, and their formation may be considered a genuine example of bacterial differentiation without visible morphological change (Figure 4).

### Phase-Variable DNA Methylation

Certain restriction-modification (R-M) systems of types I and III undergo phase variation (De Ste Croix et al., 2017; Seib et al., 2020). Switching between OFF and ON states is caused by alteration of nucleotide repeats in certain systems and by recombination in others. In some such systems, the gene encoding the restriction enzyme is inactivated by mutation, while the DNA methyltransferase gene remains active (Srikhanta et al., 2010). Phase variation of DNA methyltransferase synthesis produces two subpopulations of bacterial cells, one of which contains N^6^-methyl-adenine in the genome while the other subpopulation does not. As a consequence, each lineage shows a distinct pattern of gene expression in all DNA methylation-sensitive loci. Systems of this kind, known as phasevarions, have been described in human pathogens belonging to the genera *Haemophilus*, *Neisseria*, *Helicobacter*, *Moraxella*, *Mycoplasma*, and *Streptococcus*. Phasevarions have been shown to control envelope structure, as well as virulence and stress responses, and can facilitate immunoevasion (Srikhanta et al., 2010; Phillips et al., 2019; Seib et al., 2020).

Phasevarions are an outstanding evolutionary invention. Most phase variation systems under DNA methylation control (e.g., *pap* and *opvAB*) generate heterogeneity of a single phenotypic trait, while the cell lineages under phasevarion control differ in multiple phenotypic traits. An additional *tour de force* in the capacity of phasevarions to generate cell-to-cell diversity is found in bacterial species that produce DNA methyltransferase variants. A phasevarion of this kind controls lineage formation in the pneumococcus, *Streptococcus pneumoniae*, an opportunistic pathogen frequently found in the nasopharynx of healthy humans. The pneumococcus also causes several types of acute infection, including pneumonia and meningitis. Pneumococcal populations undergo phase variation between “opaque” and “transparent” colony phenotypes that differ in their virulence properties (Weiser et al., 1994). Subpopulations that combine traits of the two phenotypes are also produced. Formation of such lineages is under the control of a phase-variable DNA adenine methyltransferase of a type I R-M system. Six DNA methyltransferase variants are produced by site-specific recombination, and each variant generates a distinct pattern of genome methylation, which results in the formation of cell types with distinct virulence properties. Formation of such lineages may facilitate adaptation during different stages of the infection, including the crucial passage from the nasopharynx into the lung (Manso et al., 2014; Li et al., 2016; Oliver et al., 2017).

## Evolution of Phenotypic Heterogeneity

Except in obligate parasites, the biochemical machinery of prokaryotes has evolved to facilitate adaptation to changing environments. However, the adaptive capacity of a biological species is restrained by the fact that an organism can only have a limited set of traits (Maynard-Smith, 1982). Production of phenotypic variants can overcome this limitation. Because natural selection acts on phenotypes and not on genotypes, mutational and nonmutational mechanisms can be similarly suitable as sources of cell variation. An advantage of nonmutational heterogeneity is that it avoids the irreversible commitment to a new state imposed by mutation (Veening et al., 2008; Ackermann, 2015; Grimbergen et al., 2015). In fact, game theory analysis predicts that phenotypic heterogeneity can have higher adaptive value than mutation in changing environments (Kussell and Leibler, 2005; Wolf et al., 2005).

Genetic and epigenetic variations are not mutually exclusive. Nonmutational adaptation can provide a temporary window for mutation, a possibility that becomes more likely if the surviving population is large (Baquero, 2013). Hence, bacterial adaptation to new environments can be facilitated by phenotypic adaptation on a short timescale and by tuning *via* mutations in the long run (Kussell, 2013), and mutations that promote nongenetic variation (e.g., by adjusting the level of noise or the strength of a feedback loop) may be selected in a similar manner as mutations that confer any other adaptive trait.

**Figure 5:**
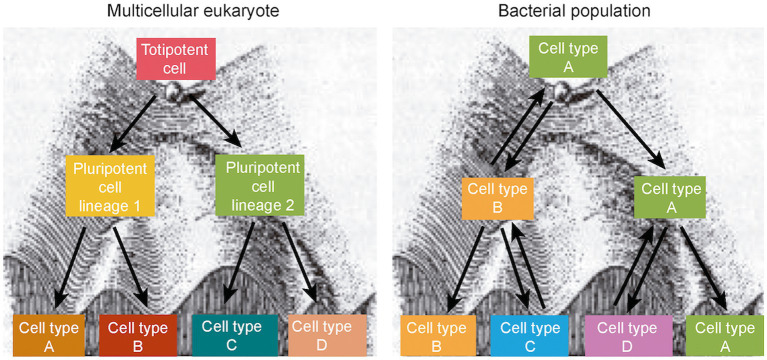
Adaptation of Waddington’s artistic drawing of an epigenetic landscape as a ball that falls to stable valleys from unstable ridges. In multicellular eukaryotes, the differentiation capacity is narrowed down at each intersection, and development proceeds in an orderly manner (left). In a bacterial epigenetic landscape, loss of differentiation capacity does not occur at any intersection, and formation of cell variants follows an aperiodic behavior typical of deterministic nonlinear systems (right).

Bacterial Waddington’s landscapes differ from their eukaryotic counterparts in a fundamental aspect, visualized in Figure 5. In a multicellular eukaryote, sequential decisions progressively curtail the cell differentiation capacity. This constraint does not exist in a bacterial population: In principle, a bacterial cell can differentiate into any other cell type. Progression through the Waddington’s landscape is thus orderly in multicellular eukaryotes and chaotic in bacteria. Unrestrained differentiation may be crucial to produce the polymorphism required for bacterial adaptation, and natural selection may tailor the level of cell-to-cell variation to the needs and challenges posed by the lifestyle of the species.

Bacterial evolution is speeded up by the fact that bacterial DNA is both somatic and germinal. As a consequence, beneficial mutations are immediately passed to the offspring without the gambles of meiosis and gamete assortment. Furthermore, the absence of canalization in bacteria exposes novel phenotypes to immediate selection (Elena and Lenski, 2001). If an adaptive phenotype is produced, natural selection will instantly act regardless of its genetic or epigenetic origin. Waddington’s landscapes can thus evolve rapidly in the bacterial world.

The capacity of a bacterial population to produce phenotypic cell variants can be appraised if one considers that independent switching of *n* bistable loci can produce 2^*n*^ types of cell variants (Sanchez-Romero et al., 2020). This theoretical number may be an overestimation as natural selection can be expected to eliminate lower fitness variants. Anyway, the actual number of phenotypic cell variants in an isogenic population of bacteria may easily surpass the detection capacity of current technologies of single-cell analysis.

## Author Contributions

MS-R and JC: conceptualization and investigation. JC: original draft preparation, administration, and funding acquisition. MS-R and JC: review and editing. Both the authors contributed to the article and approved the submitted version.

### Conflict of Interest

The authors declare that the research was conducted in the absence of any commercial or financial relationships that could be construed as a potential conflict of interest.

## References

[ref1] Ackermann M. (2015). A functional perspective on phenotypic heterogeneity in microorganisms. Nat. Rev. Microbiol. 13, 497–508. 10.1038/nrmicro3491, PMID: 26145732

[ref2] Adam M. Murali B. Glenn N. O. Potter S. S. (2008). Epigenetic inheritance based evolution of antibiotic resistance in bacteria. BMC Evol. Biol. 8:52. 10.1186/1471-2148-8-52, PMID: 18282299 PMC2262874

[ref3] Adhikari S. Curtis P. D. (2016). DNA methyltransferases and epigenetic regulation in bacteria. FEMS Microbiol. Rev. 40, 575–591. 10.1093/femsre/fuw023, PMID: 27476077

[ref4] Anderson P. W. (1972). More is different. Science 177, 393–396. 10.1126/science.177.4047.393, PMID: 17796623

[ref5] Arnoldini M. Vizcarra I. A. Pena-Miller R. Stocker N. Diard M. Vogel V.. (2014). Bistable expression of virulence genes in salmonella leads to the formation of an antibiotic-tolerant subpopulation. PLoS Biol. 12:e1001928. 10.1371/journal.pbio.1001928, PMID: 25136970 PMC4138020

[ref6] Baharoglu Z. Mazel D. (2014). SOS, the formidable strategy of bacteria against aggressions. FEMS Microbiol. Rev. 38, 1126–1145. 10.1111/1574-6976.12077, PMID: 24923554

[ref7] Balaban N. Q. (2011). Persistence: mechanisms for triggering and enhancing phenotypic variability. Curr. Opin. Genet. Dev. 21, 768–775. 10.1016/j.gde.2011.10.001, PMID: 22051606

[ref8] Balaban N. Q. Merrin J. Chait R. Kowalik L. Leibler S. (2004). Bacterial persistence as a phenotypic switch. Science 305, 1622–1625. 10.1126/science.1099390, PMID: 15308767

[ref9] Baquero F. (2013). Epigenetics, epistasis and epidemics. Evol. Med. Public Health 2013, 86–88. 10.1093/emph/eot009, PMID: 24481189 PMC3868410

[ref10] Belikova D. Jochim A. Power J. Holden M. T. G. Heilbronner S. (2020). “Gene accordions” cause genotypic and phenotypic heterogeneity in clonal populations of *Staphylococcus aureus*. Nat. Commun. 11:3526. 10.1038/s41467-020-17277-3, PMID: 32665571 PMC7360770

[ref11] Benson M. A. Lilo S. Wasserman G. A. Thoendel M. Smith A. Horswill A. R.. (2011). *Staphylococcus aureus* regulates the expression and production of the staphylococcal superantigen-like secreted proteins in a Rot-dependent manner. Mol. Microbiol. 81, 659–675. 10.1111/j.1365-2958.2011.07720.x, PMID: 21651625 PMC3217042

[ref12] Bernander R. Stokke T. Boye E. (1998). Flow cytometry of bacterial cells: comparison between different flow cytometers and different DNA stains. Cytometry 31, 29–36. 10.1002/(SICI)1097-0320(19980101)31:1<29::AID-CYTO4>3.0.CO;2-E, PMID: 9450522

[ref13] Blow M. J. Clark T. A. Daum C. G. Deutschbauer A. M. Fomenkov A. Fries R.. (2016). The epigenomic landscape of prokaryotes. PLoS Genet. 12:e1005854. 10.1371/journal.pgen.1005854, PMID: 26870957 PMC4752239

[ref14] Cambon M. C. Parthuisot N. Pages S. Lanois A. Givaudan A. Ferdy J. B. (2019). Selection of bacterial mutants in late infections: when vector transmission trades off against growth advantage in stationary phase. MBio 10:e01437-19. 10.1128/mBio.01437-19, PMID: 31594811 PMC6786866

[ref15] Cano D. A. Pucciarelli M. G. Martinez-Moya M. Casadesus J. Garcia-del Portillo F. (2003). Selection of small-colony variants of *Salmonella enterica* serovar Typhimurium in nonphagocytic eucaryotic cells. Infect. Immun. 71, 3690–3698. 10.1128/IAI.71.7.3690-3698.2003, PMID: 12819049 PMC161971

[ref16] Cao M. Goodrich-Blair H. (2020). *Xenorhabdus nematoph*ila bacteria shift from mutualistic to virulent Lrp-dependent phenotypes within the receptacles of *Steinernema carpocapsae* insect-infective stage nematodes. Environ. Microbiol. 22, 5433–5449. 10.1111/1462-2920.15286, PMID: 33078552

[ref17] Casadesus J. D’Ari R. (2002). Memory in bacteria and phage. BioEssays 24, 512–518. 10.1002/bies.10102, PMID: 12111734

[ref18] Casadesus J. Low D. (2006). Epigenetic gene regulation in the bacterial world. Microbiol. Mol. Biol. Rev. 70, 830–856. 10.1128/MMBR.00016-06, PMID: 16959970 PMC1594586

[ref19] Casadesus J. Low D. A. (2013). Programmed heterogeneity: epigenetic mechanisms in bacteria. J. Biol. Chem. 288, 13929–13935. 10.1074/jbc.R113.472274, PMID: 23592777 PMC3656251

[ref20] Casadesus J. Torreblanca J. (1996). “Methylation-related epigenetic signals in bacterial DNA” in Epigenetic Mechanisms of Gene Regulation. eds. Russo V. E. A. Martienssen R. A. Riggs A. D. (Cold Spring Harbor, New York: Cold Spring Harbor Laboratory), 141–153.

[ref21] Claudi B. Sprote P. Chirkova A. Personnic N. Zankl J. Schurmann N.. (2014). Phenotypic variation of *Salmonella* in host tissues delays eradication by antimicrobial chemotherapy. Cell 158, 722–733. 10.1016/j.cell.2014.06.045, PMID: 25126781

[ref22] Collier J. (2019). Cell division control in *Caulobacter crescentus*. Biochim. Biophys. Acta Gene Regul. Mech. 1862, 685–690. 10.1016/j.bbagrm.2018.04.005, PMID: 29715525

[ref23] Cota I. Sanchez-Romero M. A. Hernandez S. B. Pucciarelli M. G. Garcia-Del Portillo F. Casadesus J. (2015). Epigenetic control of *Salmonella enterica* O-antigen chain length: a tradeoff between virulence and bacteriophage resistance. PLoS Genet. 11:e1005667. 10.1371/journal.pgen.1005667, PMID: 26583926 PMC4652898

[ref24] Crawford R. W. Rosales-Reyes R. Ramirez-Aguilar Mde L. Chapa-Azuela O. Alpuche-Aranda C. Gunn J. S. (2010). Gallstones play a significant role in *Salmonella* spp. gallbladder colonization and carriage. Proc. Natl. Acad. Sci. U. S. A. 107, 4353–4358. 10.1073/pnas.1000862107, PMID: 20176950 PMC2840110

[ref25] Dandach S. H. Khammash M. (2010). Analysis of stochastic strategies in bacterial competence: a master equation approach. PLoS Comp. Biol. 6:e1000985. 10.1371/journal.pcbi.1000985, PMID: 21085679 PMC2978674

[ref26] Davis B. M. Chao M. C. Waldor M. K. (2013). Entering the era of bacterial epigenomics with single molecule real time DNA sequencing. Curr. Opin. Microbiol. 16, 192–198. 10.1016/j.mib.2013.01.011, PMID: 23434113 PMC3646917

[ref27] de Jong I. G. Haccou P. Kuipers O. P. (2011). Bet hedging or not? A guide to proper classification of microbial survival strategies. BioEssays 33, 215–223. 10.1002/bies.201000127, PMID: 21254151

[ref28] De Ste Croix M. Vacca I. Kwun M. J. Ralph J. D. Bentley S. D. Haigh R.. (2017). Phase-variable methylation and epigenetic regulation by type I restriction-modification systems. FEMS Microbiol. Rev. 41(Suppl. 1), S3–S15. 10.1093/femsre/fux025, PMID: 28830092

[ref29] Dewachter L. Fauvart M. Michiels J. (2019). Bacterial heterogeneity and antibiotic survival: understanding and combatting persistence and heteroresistance. Mol. Cell 76, 255–267. 10.1016/j.molcel.2019.09.028, PMID: 31626749

[ref30] Dhar N. McKinney J. D. (2007). Microbial phenotypic heterogeneity and antibiotic tolerance. Curr. Opin. Microbiol. 10, 30–38. 10.1016/j.mib.2006.12.007, PMID: 17215163

[ref31] Diard M. Garcia V. Maier L. Remus-Emsermann M. N. Regoes R. R. Ackermann M.. (2013). Stabilization of cooperative virulence by the expression of an avirulent phenotype. Nature 494, 353–356. 10.1038/nature11913, PMID: 23426324

[ref32] Dragos A. Kiesewalter H. Martin M. Hsu C. Y. Hartmann R. Wechsler T.. (2018). Division of labor during biofilm matrix production. Curr. Biol. 28, 1903–1913. 10.1016/j.cub.2018.04.046, PMID: 29887307 PMC6331042

[ref33] Dubnau D. Losick R. (2006). Bistability in bacteria. Mol. Microbiol. 61, 564–572. 10.1111/j.1365-2958.2006.05249.x, PMID: 16879639

[ref34] El Meouche I. Siu Y. Dunlop M. J. (2016). Stochastic expression of a multiple antibiotic resistance activator confers transient resistance in single cells. Sci. Rep. 6:19538. 10.1038/srep19538, PMID: 26758525 PMC4725842

[ref35] Elena S. F. Lenski R. E. (2001). Epistasis between new mutations and genetic background and a test of genetic canalization. Evolution 55, 1746–1752. 10.1111/j.0014-3820.2001.tb00824.x, PMID: 11681730

[ref36] Ferrell J. E. Jr. (2002). Self-perpetuating states in signal transduction: positive feedback, double-negative feedback and bistability. Curr. Opin. Cell Biol. 14, 140–148. 10.1016/S0955-0674(02)00314-9, PMID: 11891111

[ref37] Finlay B. B. McFadden G. (2006). Anti-immunology: evasion of the host immune system by bacterial and viral pathogens. Cell 124, 767–782. 10.1016/j.cell.2006.01.034, PMID: 16497587

[ref38] Fisher R. A. Gollan B. Helaine S. (2017). Persistent bacterial infections and persister cells. Nat. Rev. Microbiol. 15, 453–464. 10.1038/nrmicro.2017.42, PMID: 28529326

[ref39] Fitch W. M. (1982). The challenges to Darwinism since the last centennial and the impact of molecular studies. Evolution 36, 1133–1143. 10.1111/j.1558-5646.1982.tb05484.x, PMID: 28563562

[ref40] Flusberg B. A. Webster D. R. Lee J. H. Travers K. J. Olivares E. C. Clark T. A.. (2010). Direct detection of DNA methylation during single-molecule, real-time sequencing. Nat. Methods 7, 461–465. 10.1038/nmeth.1459, PMID: 20453866 PMC2879396

[ref41] García-Betancur J. C. Goñi-Moreno A. Horger T. Schott M. Sharan M. Eikmeier J.. (2017). Cell differentiation defines acute and chronic infection cell types in *Staphylococcus aureus*. eLife 6:e28023. 10.7554/eLife.28023, PMID: 28893374 PMC5595439

[ref42] Garcia-Del Portillo F. (2008). Heterogeneity in tissue culture infection models: a source of novel host-pathogen interactions? Microbes Infect. 10, 1063–1066. 10.1016/j.micinf.2008.07.004, PMID: 18662799

[ref43] Garcia-Pastor L. Sanchez-Romero M. A. Gutierrez G. Puerta-Fernandez E. Casadesus J. (2018). Formation of phenotypic lineages in *Salmonella enterica* by a pleiotropic fimbrial switch. PLoS Genet. 14:e1007677. 10.1371/journal.pgen.1007677, PMID: 30252837 PMC6173445

[ref44] Gillespie J. H. (1974). Natural selection for within-generation variance in offspring number. Genetics 76, 601–606. 10.1093/genetics/76.3.601, PMID: 4833578 PMC1213089

[ref45] Gordon A. J. Halliday J. A. Blankschien M. D. Burns P. A. Yatagai F. Herman C. (2009). Transcriptional infidelity promotes heritable phenotypic change in a bistable gene network. PLoS Biol. 7:e44. 10.1371/journal.pbio.1000044, PMID: 19243224 PMC2652393

[ref46] Grimbergen A. J. Siebring J. Solopova A. Kuipers O. P. (2015). Microbial bet-hedging: the power of being different. Curr. Opin. Microbiol. 25, 67–72. 10.1016/j.mib.2015.04.008, PMID: 26025019

[ref47] Hamoen L. W. Kausche D. Marahiel M. A. van Sinderen D. Venema G. Serror P. (2003). The *Bacillus subtilis* transition state regulator AbrB binds to the −35 promoter region of *comK*. FEMS Microbiol. Lett. 218, 299–304. 10.1111/j.1574-6968.2003.tb11532.x, PMID: 12586407

[ref48] Helaine S. Thompson J. A. Watson K. G. Liu M. Boyle C. Holden D. W. (2010). Dynamics of intracellular bacterial replication at the single cell level. Proc. Natl. Acad. Sci. U. S. A. 107, 3746–3751. 10.1073/pnas.1000041107, PMID: 20133586 PMC2840444

[ref49] Hernandez S. B. Cota I. Ducret A. Aussel L. Casadesus J. (2012). Adaptation and preadaptation of *Salmonella enterica* to bile. PLoS Genet. 8:e1002459. 10.1371/journal.pgen.1002459, PMID: 22275872 PMC3261920

[ref50] Hernday A. Braaten B. Low D. (2004). The intricate workings of a bacterial epigenetic switch. Adv. Exp. Med. Biol. 547, 83–89. 10.1007/978-1-4419-8861-4_7 15230094

[ref51] Hoa T. T. Tortosa P. Albano M. Dubnau D. (2002). Rok (YkuW) regulates genetic competence in *Bacillus subtilis* by directly repressing comK. Mol. Microbiol. 43, 15–26. 10.1046/j.1365-2958.2002.02727.x, PMID: 11849533

[ref52] Hofsteenge N. van Nimwegen E. Silander O. K. (2013). Quantitative analysis of persister fractions suggests different mechanisms of formation among environmental isolates of *E. coli*. BMC Microbiol. 13:25. 10.1186/1471-2180-13-25, PMID: 23379956 PMC3682893

[ref53] Hussa E. A. Casanova-Torres A. M. Goodrich-Blair H. (2015). The global transcription factor Lrp controls virulence modulation in *Xenorhabdus nematophila*. J. Bacteriol. 197, 3015–3025. 10.1128/JB.00272-15, PMID: 26170407 PMC4542165

[ref54] Imamovic L. Balleste E. Martinez-Castillo A. Garcia-Aljaro C. Muniesa M. (2016). Heterogeneity in phage induction enables the survival of the lysogenic population. Environ. Microbiol. 18, 957–969. 10.1111/1462-2920.13151, PMID: 26626855

[ref55] Johnson A. D. Poteete A. R. Lauer G. Sauer R. T. Ackers G. K. Ptashne M. (1981). Lambda repressor and cro – components of an efficient molecular switch. Nature 294, 217–223. 10.1038/294217a0, PMID: 6457992

[ref56] Kaern M. Elston T. C. Blake W. J. Collins J. J. (2005). Stochasticity in gene expression: from theories to phenotypes. Nat. Rev. Genet. 6, 451–464. 10.1038/nrg1615, PMID: 15883588

[ref57] Kamensek S. Podlesek Z. Gillor O. Zgur-Bertok D. (2010). Genes regulated by the *Escherichia coli* SOS repressor LexA exhibit heterogeneous expression. BMC Microbiol. 10:283. 10.1186/1471-2180-10-283, PMID: 21070632 PMC2994835

[ref58] Khanna K. Lopez-Garrido J. Pogliano K. (2020). Shaping an endospore: architectural transformations during *Bacillus subtilis* sporulation. Annu. Rev. Microbiol. 74, 361–386. 10.1146/annurev-micro-022520-074650, PMID: 32660383 PMC7610358

[ref59] Kondorosi E. Mergaert P. Kereszt A. (2013). A paradigm for endosymbiotic life: cell differentiation of *Rhizobium* bacteria provoked by host plant factors. Annu. Rev. Microbiol. 67, 611–628. 10.1146/annurev-micro-092412-155630, PMID: 24024639

[ref60] Kreibich S. Hardt W. D. (2015). Experimental approaches to phenotypic diversity in infection. Curr. Opin. Microbiol. 27, 25–36. 10.1016/j.mib.2015.06.007, PMID: 26143306

[ref61] Kumar R. Rao D. N. (2013). Role of DNA methyltransferases in epigenetic regulation in bacteria. Subcell. Biochem. 61, 81–102. 10.1007/978-94-007-4525-4_4 23150247

[ref62] Kussell E. (2013). Evolution in microbes. Annu. Rev. Biophys. 42, 493–514. 10.1146/annurev-biophys-083012-130320, PMID: 23654305

[ref63] Kussell E. Leibler S. (2005). Phenotypic diversity, population growth, and information in fluctuating environments. Science 309, 2075–2078. 10.1126/science.1114383, PMID: 16123265

[ref64] Lambert G. Kussell E. (2014). Memory and fitness optimization of bacteria under fluctuating environments. PLoS Genet. 10:e1004556. 10.1371/journal.pgen.1004556, PMID: 25255314 PMC4177670

[ref65] Laurent M. Charvin G. Guespin-Michel J. (2005). Bistability and hysteresis in epigenetic regulation of the lactose operon. Since Delbruck, a long series of ignored models. Cell. Mol. Biol. 51, 583–594. 16359608

[ref66] Leigh E. G. Jr. (2010). The group selection controversy. J. Evol. Biol. 23, 6–19. 10.1111/j.1420-9101.2009.01876.x, PMID: 20002254

[ref67] Li J. Li J. W. Feng Z. Wang J. An H. Liu Y.. (2016). Epigenetic switch driven by DNA inversions dictates phase variation in *Streptococcus pneumoniae*. PLoS Pathog. 12:e1005762. 10.1371/journal.ppat.1005762, PMID: 27427949 PMC4948785

[ref68] Lobner-Olesen A. Skovgaard O. Marinus M. G. (2005). Dam methylation: coordinating cellular processes. Curr. Opin. Microbiol. 8, 154–160. 10.1016/j.mib.2005.02.009, PMID: 15802246

[ref69] Low D. A. Casadesus J. (2008). Clocks and switches: bacterial gene regulation by DNA adenine methylation. Curr. Opin. Microbiol. 11, 106–112. 10.1016/j.mib.2008.02.012, PMID: 18396448

[ref70] Magnuson R. Solomon J. Grossman A. D. (1994). Biochemical and genetic characterization of a competence pheromone from *B. subtilis*. Cell 77, 207–216. 10.1016/0092-8674(94)90313-1, PMID: 8168130

[ref71] Manina G. Griego A. Singh L. K. McKinney J. D. Dhar N. (2019). Preexisting variation in DNA damage response predicts the fate of single mycobacteria under stress. EMBO J. 38:e101876. 10.15252/embj.2019101876, PMID: 31583725 PMC6856624

[ref72] Manso A. S. Chai M. H. Atack J. M. Furi L. De Ste Croix M. Haigh R.. (2014). A random six-phase switch regulates pneumococcal virulence via global epigenetic changes. Nat. Commun. 5:6055. 10.1038/ncomms6055, PMID: 25268848 PMC4190663

[ref73] Marinus M. G. (1996). “Methylation of DNA” in Escherichia coli and Salmonella: Cellular and Molecular Biology. eds. Neidhardt F. C. Curtiss R. Ingraham J. L. Lin E. C. C. Low K. B. Magasanik B.. (Washington, DC: ASM Press), 782–791.

[ref74] Marinus M. G. Casadesus J. (2009). Roles of DNA adenine methylation in host-pathogen interactions: mismatch repair, transcriptional regulation, and more. FEMS Microbiol. Rev. 33, 488–503. 10.1111/j.1574-6976.2008.00159.x, PMID: 19175412 PMC2941194

[ref75] Maynard-Smith J. (1982). Evolution and the Theory of Games. Cambridge, England: Cambridge University Press.

[ref76] McCool J. D. Long E. Petrosino J. F. Sandler H. A. Rosenberg S. M. Sandler S. J. (2004). Measurement of SOS expression in individual *Escherichia coli* K-12 cells using fluorescence microscopy. Mol. Microbiol. 53, 1343–1357. 10.1111/j.1365-2958.2004.04225.x, PMID: 15387814

[ref77] Menendez A. Arena E. T. Guttman J. A. Thorson L. Vallance B. A. Vogl W.. (2009). *Salmonella* infection of gallbladder epithelial cells drives local inflammation and injury in a model of acute typhoid fever. J. Infect. Dis. 200, 1703–1713. 10.1086/646608, PMID: 19852670

[ref78] Meyer P. Dworkin J. (2007). Applications of fluorescence microscopy to single bacterial cells. Res. Microbiol. 158, 187–194. 10.1016/j.resmic.2006.12.008, PMID: 17349779

[ref79] Motta S. S. Cluzel P. Aldana M. (2015). Adaptive resistance in bacteria requires epigenetic inheritance, genetic noise, and cost of efflux pumps. PLoS One 10:e0118464. 10.1371/journal.pone.0118464, PMID: 25781931 PMC4363326

[ref80] Mouammine A. Collier J. (2018). The impact of DNA methylation in *Alphaproteobacteria*. Mol. Microbiol. 110, 1–10. 10.1111/mmi.14079, PMID: 29995343

[ref81] Moxon R. Bayliss C. Hood D. (2006). Bacterial contingency loci: the role of simple sequence DNA repeats in bacterial adaptation. Annu. Rev. Genet. 40, 307–333. 10.1146/annurev.genet.40.110405.090442, PMID: 17094739

[ref82] Muller J. Spriewald S. Stecher B. Stadler E. Fuchs T. M. (2019). Evolutionary stability of *Salmonella* competition with the gut microbiota: how the environment fosters heterogeneity in exploitative and interference competition. J. Mol. Biol. 431, 4732–4748. 10.1016/j.jmb.2019.06.027, PMID: 31260689

[ref83] Munoz-Dorado J. Marcos-Torres F. J. Garcia-Bravo E. Moraleda-Munoz A. Perez J. (2016). Myxobacteria: moving, killing, feeding, and surviving together. Front. Microbiol. 7:781. 10.3389/fmicb.2016.00781, PMID: 27303375 PMC4880591

[ref84] Munsky B. Khammash M. (2010). Identification from stochastic cell-to-cell variation: a genetic switch case study. IET Syst. Biol. 4, 356–366. 10.1049/iet-syb.2010.0013, PMID: 21073235

[ref85] Muro-Pastor A. M. Hess W. R. (2012). Heterocyst differentiation: from single mutants to global approaches. Trends Microbiol. 20, 548–557. 10.1016/j.tim.2012.07.005, PMID: 22898147

[ref86] Nelson L. K. Stanton M. M. Elphinstone R. E. A. Helwerda J. Turner R. J. Ceri H. (2010). Phenotypic diversification in vivo: *Pseudomonas aeruginosa* gacS- strains generate small colony variants in vivo that are distinct from in vitro variants. Microbiology 156, 3699–3709. 10.1099/mic.0.040824-0 20817643

[ref87] Nielsen A. T. Dolganov N. A. Rasmussen T. Otto G. Miller M. C. Felt S. A.. (2010). A bistable switch and anatomical site control vibrio cholerae virulence gene expression in the intestine. PLoS Pathog. 6:e1001102. 10.1371/journal.ppat.1001102, PMID: 20862321 PMC2940755

[ref88] Novick A. Weiner M. (1957). Enzyme induction as an all-or-none phenomenon. Proc. Natl. Acad. Sci. U. S. A. 43, 553–566. 16590055 10.1073/pnas.43.7.553 PMC528498

[ref89] Nuss A. M. Schuster F. Roselius L. Klein J. Bucker R. Herbst K.. (2016). A precise temperature-responsive bistable switch controlling *Yersinia* virulence. PLoS Pathog. 12:e1006091. 10.1371/journal.ppat.1006091, PMID: 28006011 PMC5179001

[ref90] Oliver A. Canton R. Campo P. Baquero F. Blazquez J. (2000). High frequency of hypermutable *Pseudomonas aeruginosa* in cystic fibrosis lung infection. Science 288, 1251–1254. 10.1126/science.288.5469.1251, PMID: 10818002

[ref91] Oliver M. B. Basu Roy A. Kumar R. Lefkowitz E. J. Swords W. E. (2017). *Streptococcus pneumoniae* TIGR4 phase-locked opacity variants differ in virulence phenotypes. mSphere 2:e00386-17. 10.1128/mSphere.00386-17, PMID: 29152579 PMC5687919

[ref92] Pennington J. M. Rosenberg S. M. (2007). Spontaneous DNA breakage in single living *Escherichia coli* cells. Nat. Genet. 39, 797–802. 10.1038/ng2051, PMID: 17529976 PMC2856310

[ref93] Phillips Z. N. Husna A. U. Jennings M. P. Seib K. L. Atack J. M. (2019). Phasevarions of bacterial pathogens - phase-variable epigenetic regulators evolving from restriction-modification systems. Microbiology 165, 917–928. 10.1099/mic.0.000805, PMID: 30994440

[ref94] Pribis J. P. Garcia-Villada L. Zhai Y. Lewin-Epstein O. Wang A. Z. Liu J.. (2019). Gamblers: an antibiotic-induced evolvable cell subpopulation differentiated by reactive-oxygen-induced general stress response. Mol. Cell 74, 785–800. 10.1016/j.molcel.2019.02.037, PMID: 30948267 PMC6553487

[ref95] Proctor R. A. Balwit J. M. Vesga O. (1994). Variant subpopulations of *Staphylococcus aureus* as cause of persistent and recurrent infections. Infect. Agents Dis. 3, 302–312. PMID: 7889317

[ref96] Prouty A. M. Schwesinger W. H. Gunn J. S. (2002). Biofilm formation and interaction with the surfaces of gallstones by *Salmonella* spp. Infect. Immun. 70, 2640–2649. 10.1128/IAI.70.5.2640-2649.2002, PMID: 11953406 PMC127943

[ref97] Putrins M. Kogermann K. Lukk E. Lippus M. Varik V. Tenson T. (2015). Phenotypic heterogeneity enables uropathogenic *Escherichia coli* to evade killing by antibiotics and serum complement. Infect. Immun. 83, 1056–1067. 10.1128/IAI.02725-14, PMID: 25561706 PMC4333455

[ref98] Recsei P. Kreiswirth B. O’Reilly M. Schlievert P. Gruss A. Novick R. P. (1986). Regulation of exoprotein gene expression in *Staphylococcus aureus* by agar. Mol. Gen. Genet. 202, 58–61. 10.1007/BF00330517, PMID: 3007938

[ref99] Reyes Ruiz L. M. Williams C. L. Tamayo R. (2020). Enhancing bacterial survival through phenotypic heterogeneity. PLoS Pathog. 16:e1008439. 10.1371/journal.ppat.1008439, PMID: 32437427 PMC7241687

[ref100] Rhen M. Eriksson S. Clements M. Bergstrom S. Normark S. J. (2003). The basis of persistent bacterial infections. Trends Microbiol. 11, 80–86. 10.1016/S0966-842X(02)00038-0, PMID: 12598130

[ref101] Roth J. R. Kugelberg E. Reams A. B. Kofoid E. Andersson D. I. (2006). Origin of mutations under selection: the adaptive mutation controversy. Annu. Rev. Microbiol. 60, 477–501. 10.1146/annurev.micro.60.080805.142045, PMID: 16761951

[ref102] Saint-Ruf C. Matic I. (2006). Environmental tuning of mutation rates. Environ. Microbiol. 8, 193–199. 10.1046/j.1462-2920.2003.00397.x-i1, PMID: 16423008

[ref103] Sanchez A. Choubey S. Kondev J. (2013). Regulation of noise in gene expression. Annu. Rev. Biophys. 42, 469–491. 10.1146/annurev-biophys-083012-130401, PMID: 23527780

[ref104] Sanchez-Romero M. A. Casadesus J. (2014). Contribution of phenotypic heterogeneity to adaptive antibiotic resistance. Proc. Natl. Acad. Sci. U. S. A. 111, 355–360. 10.1073/pnas.1316084111, PMID: 24351930 PMC3890857

[ref105] Sanchez-Romero M. A. Casadesus J. (2018). Contribution of SPI-1 bistability to *Salmonella enterica* cooperative virulence: insights from single cell analysis. Sci. Rep. 8:14875. 10.1038/s41598-018-33137-z, PMID: 30291285 PMC6173691

[ref106] Sanchez-Romero M. A. Casadesus J. (2020). The bacterial epigenome. Nat. Rev. Microbiol. 18, 7–20. 10.1038/s41579-019-0286-2, PMID: 31728064

[ref107] Sanchez-Romero M. A. Olivenza D. R. Gutierrez G. Casadesus J. (2020). Contribution of DNA adenine methylation to gene expression heterogeneity in *Salmonella enterica*. Nucleic Acids Res. 48, 11857–11867. 10.1093/nar/gkaa730, PMID: 32954419 PMC7708049

[ref108] Satory D. Gordon A. J. Halliday J. A. Herman C. (2011). Epigenetic switches: can infidelity govern fate in microbes? Curr. Opin. Microbiol. 14, 212–217. 10.1016/j.mib.2010.12.004, PMID: 21496764

[ref109] Scheler O. Postek W. Garstecki P. (2019). Recent developments of microfluidics as a tool for biotechnology and microbiology. Curr. Opin. Biotechnol. 55, 60–67. 10.1016/j.copbio.2018.08.004, PMID: 30172910

[ref110] Schreiber F. Littmann S. Lavik G. Escrig S. Meibom A. Kuypers M. M.. (2016). Phenotypic heterogeneity driven by nutrient limitation promotes growth in fluctuating environments. Nat. Microbiol. 1:16055. 10.1038/nmicrobiol.2016.55, PMID: 27572840

[ref111] Schroter L. Dersch P. (2019). Phenotypic diversification of microbial pathogens -cooperating and preparing for the future. J. Mol. Biol. 431, 4645–4655. 10.1016/j.jmb.2019.06.024, PMID: 31260693

[ref112] Scott T. N. Simon M. I. (1982). Genetic analysis of the mechanism of the *Salmonella* phase variation site specific recombination system. Mol. Gen. Genet. 188, 313–321. 10.1007/BF00332694, PMID: 6759874

[ref113] Seib K. L. Srikhanta Y. N. Atack J. M. Jennings M. P. (2020). Epigenetic regulation of virulence and immunoevasion by phase-variable restriction-modification systems in bacterial pathogens. Annu. Rev. Microbiol. 74, 655–671. 10.1146/annurev-micro-090817-062346, PMID: 32689914

[ref114] Shapiro J. A. (1998). Thinking about bacterial populations as multicellular organisms. Annu. Rev. Microbiol. 52, 81–104. 10.1146/annurev.micro.52.1.81, PMID: 9891794

[ref115] Silva-Rocha R. de Lorenzo V. (2010). Noise and robustness in prokaryotic regulatory networks. Annu. Rev. Microbiol. 64, 257–275. 10.1146/annurev.micro.091208.073229, PMID: 20825349

[ref116] Smits W. K. Eschevins C. C. Susanna K. A. Bron S. Kuipers O. P. Hamoen L. W. (2005). Stripping bacillus: ComK auto-stimulation is responsible for the bistable response in competence development. Mol. Microbiol. 56, 604–614. 10.1111/j.1365-2958.2005.04488.x, PMID: 15819618

[ref117] Smits W. K. Kuipers O. P. Veening J. W. (2006). Phenotypic variation in bacteria: the role of feedback regulation. Nat. Rev. Microbiol. 4, 259–271. 10.1038/nrmicro1381, PMID: 16541134

[ref118] Srikhanta Y. N. Fox K. L. Jennings M. P. (2010). The phasevarion: phase variation of type III DNA methyltransferases controls coordinated switching in multiple genes. Nat. Rev. Microbiol. 8, 196–206. 10.1038/nrmicro2283, PMID: 20140025

[ref119] Sturm A. Heinemann M. Arnoldini M. Benecke A. Ackermann M. Benz M.. (2011). The cost of virulence: retarded growth of *Salmonella* Typhimurium cells expressing type III secretion system 1. PLoS Pathog. 7:e1002143. 10.1371/journal.ppat.1002143, PMID: 21829349 PMC3145796

[ref120] Suwandi A. Galeev A. Riedel R. Sharma S. Seeger K. Sterzenbach T.. (2019). Std fimbriae-fucose interaction increases *Salmonella*-induced intestinal inflammation and prolongs colonization. PLoS Pathog. 15:e1007915. 10.1371/journal.ppat.1007915, PMID: 31329635 PMC6675130

[ref121] Thomas R. Kaufman M. (2001). Multistationarity, the basis of cell differentiation and memory. II. Logical analysis of regulatory networks in terms of feedback circuits. Chaos 11, 180–195. 10.1063/1.1349893, PMID: 12779452

[ref122] Tomanek I. Grah R. Lagator M. Andersson A. M. C. Bollback J. P. Tkacik G.. (2020). Gene amplification as a form of population-level gene expression regulation. Nat. Ecol. Evol. 4, 612–625. 10.1038/s41559-020-1132-7, PMID: 32152532

[ref123] Turgay K. Hahn J. Burghoorn J. Dubnau D. (1998). Competence in *Bacillus subtilis* is controlled by regulated proteolysis of a transcription factor. EMBO J. 17, 6730–6738. 10.1093/emboj/17.22.6730, PMID: 9890793 PMC1171018

[ref124] Turkington C. J. R. Morozov A. Clokie M. R. J. Bayliss C. D. (2019). Phage-resistant phase-variant sub-populations mediate herd immunity against bacteriophage invasion of bacterial meta-populations. Front. Microbiol. 10:1473. 10.3389/fmicb.2019.01473 31333609 PMC6625227

[ref125] Turner K. H. Vallet-Gely I. Dove S. L. (2009). Epigenetic control of virulence gene expression in *Pseudomonas aeruginosa* by a LysR-type transcription regulator. PLoS Genet. 5:e1000779. 10.1371/journal.pgen.1000779, PMID: 20041030 PMC2796861

[ref126] Uphoff S. Lord N. D. Okumus B. Potvin-Trottier L. Sherratt D. J. Paulsson J. (2016). Stochastic activation of a DNA damage response causes cell-to-cell mutation rate variation. Science 351, 1094–1097. 10.1126/science.aac9786, PMID: 26941321 PMC4827329

[ref127] Urdaneta V. Casadesus J. (2017). Interactions between bacteria and bile salts in the gastrointestinal and hepatobiliary tracts. Front. Med. 4:163. 10.3389/fmed.2017.00163, PMID: 29043249 PMC5632352

[ref128] Urdaneta V. Hernandez S. B. Casadesus J. (2019). Mutational and non mutational adaptation o*f Salmonella enterica* to the gall bladder. Sci. Rep. 9:5203. 10.1038/s41598-019-41600-8, PMID: 30914708 PMC6435676

[ref129] van der Woude M. Braaten B. Low D. (1996). Epigenetic phase variation of the *pap* operon in *Escherichia coli*. Trends Microbiol. 4, 5–9. 10.1016/0966-842X(96)81498-3, PMID: 8824788

[ref130] van der Woude M. W. (2011). Phase variation: how to create and coordinate population diversity. Curr. Opin. Microbiol. 14, 205–211. 10.1016/j.mib.2011.01.002, PMID: 21292543

[ref131] van Gestel J. Vlamakis H. Kolter R. (2015). Division of labor in biofilms: the ecology of cell differentiation. Microbiol. Spectr. 3:MB-0002-2014. 10.1128/microbiolspec.MB-0002-2014, PMID: 26104716

[ref132] van Sinderen D. Luttinger A. Kong L. Dubnau D. Venema G. Hamoen L. (1995). *comK* encodes the competence transcription factor, the key regulatory protein for competence development in *Bacillus subtilis*. Mol. Microbiol. 15, 455–462. 10.1111/j.1365-2958.1995.tb02259.x, PMID: 7783616

[ref133] Vasu K. Nagaraja V. (2013). Diverse functions of restriction-modification systems in addition to cellular defense. Microbiol. Mol. Biol. Rev. 77, 53–72. 10.1128/MMBR.00044-12, PMID: 23471617 PMC3591985

[ref134] Veening J. W. Smits W. K. Kuipers O. P. (2008). Bistability, epigenetics, and bet-hedging in bacteria. Annu. Rev. Microbiol. 62, 193–210. 10.1146/annurev.micro.62.081307.163002, PMID: 18537474

[ref135] Vincent M. S. Uphoff S. (2020). Bacterial phenotypic heterogeneity in DNA repair and mutagenesis. Biochem. Soc. Trans. 48, 451–462. 10.1042/BST20190364, PMID: 32196548 PMC7200632

[ref136] Waddington C. H. (1957). The Strategy of the Genes. London: George Allen and Unwin.

[ref137] Weigel W. A. Dersch P. (2018). Phenotypic heterogeneity: a bacterial virulence strategy. Microbes Infect. 20, 570–577. 10.1016/j.micinf.2018.01.008, PMID: 29409898

[ref138] Weiser J. N. Austrian R. Sreenivasan P. K. Masure H. R. (1994). Phase variation in pneumococcal opacity: relationship between colonial morphology and nasopharyngeal colonization. Infect. Immun. 62, 2582–2589. 10.1128/IAI.62.6.2582-2589.1994, PMID: 8188381 PMC186548

[ref139] Wion D. Casadesus J. (2006). N^6^-methyl-adenine: an epigenetic signal for DNA-protein interactions. Nat. Rev. Microbiol. 4, 183–192. 10.1038/nrmicro1350, PMID: 16489347 PMC2755769

[ref140] Wolf D. M. Vazirani V. V. Arkin A. P. (2005). Diversity in times of adversity: probabilistic strategies in microbial survival games. J. Theor. Biol. 234, 227–253. 10.1016/j.jtbi.2004.11.020, PMID: 15757681

[ref141] Woods E. C. McBride S. M. (2017). Regulation of antimicrobial resistance by extracytoplasmic function (ECF) sigma factors. Microbes Infect. 19, 238–248. 10.1016/j.micinf.2017.01.007, PMID: 28153747 PMC5403605

[ref142] Xia J. Chiu L. Y. Nehring R. B. Bravo Nunez M. A. Mei Q. Perez M.. (2019). Bacteria-to-human protein networks reveal origins of endogenous DNA damage. Cell 176, 127–143.e124. 10.1016/j.cell.2018.12.008, PMID: 30633903 PMC6344048

[ref143] Yang L. Mih N. Anand A. Park J. H. Tan J. Yurkovich J. T.. (2019). Cellular responses to reactive oxygen species are predicted from molecular mechanisms. Proc. Natl. Acad. Sci. U. S. A. 116, 14368–14373. 10.1073/pnas.1905039116, PMID: 31270234 PMC6628673

[ref144] Zhang Z. Claessen D. Rozen D. E. (2016). Understanding microbial divisions of labor. Front. Microbiol. 7:2070. 10.3389/fmicb.2016.02070, PMID: 28066387 PMC5174093

